# Prevalence and risk factors of myopia in adult Korean population: Korea national health and nutrition examination survey 2013-2014 (KNHANES VI)

**DOI:** 10.1371/journal.pone.0211204

**Published:** 2019-01-24

**Authors:** Sang Beom Han, Jieun Jang, Hee Kyung Yang, Jeong-Min Hwang, Sue K. Park

**Affiliations:** 1 Department of Ophthalmology, Kangwon National University Hospital, Kangwon National University Graduate School of Medicine, Chuncheon, Korea; 2 Department of Preventive Medicine, Seoul National University College of Medicine, Seoul, Korea; 3 Department of Ophthalmology, Seoul National University College of Medicine, Seoul, Korea; National Yang-Ming University Hospital, TAIWAN

## Abstract

**Purpose:**

To evaluate the prevalence and risk factors of myopia in adult Korean population.

**Methods:**

Population-based cross-sectional data of 3,398 subjects aged 19 to 49 years was obtained using the Korea National Health and Nutrition Examination Survey 2013–2014 (KNHANES VI). Data, including refractive errors and potential risk factors were analyzed. The prevalence and risk factors of myopia, low myopia, and high myopia—defined as a spherical equivalent (SEQ) ≤ -0.5 diopters (D), -6.0 D < SEQ <-0.5 D, and SEQ ≤ -6.0 D, respectively—were evaluated.

**Results:**

The prevalence of myopia and high myopia were 70.6 (standard error (SE), ±1.1)% and 8.0 (SE, ±0.6)%, respectively. In multivariable analysis, younger age, higher education (≥12 years), parental myopia, lower serum 25-hydroxyvitamin D (25(OH)D) concentration (<9 ng/mL), longer time spent on near work (≥3 hours/day), and higher white blood cell (WBC) count (5–8.9 x 10^3^) were associated with increased prevalence of both myopia and high myopia. Serum 25(OH)D concentration of ≥ 9 ng/ml was significantly associated with decreased prevalence of high myopia in participants with near work of ≥3 hours/day, although the effect was not significant in myopia and low myopia.

**Conclusions:**

The prevalence of myopia and high myopia in Korean adults was substantially high, which increased with decreasing age. In addition to parental myopia, the serum 25(OH)D concentration, near work and inflammation reflected by WBC counts may be associated with myopia.

## Introduction

Myopia is one of the most common ophthalmologic disorders and a major public health concern worldwide [1–3]. The prevalence of myopia varies depending on ethnicity, region, and age group [4, 5]. A meta-analysis estimated that the crude prevalence rates for myopia of spherical equivalent (SEQ) ≤ -1.0 D ranged from 16.4%–26.6% among those aged ≥40 years in the United States, Western Europe, and Australia [6]. The prevalence of myopia of ≤ -0.5 D in East Asians ≥40 years was 26.2%–41.8% [7–10], which is substantially higher compared with Caucasians. Myopia prevalence has sharply increased in the younger generation in East Asia [5, 11–13]. It was reported to be as high as 79.3% in Singapore military conscripts aged 17–19 years, and 86.1% in Taiwan conscripts aged 18–24 years [5, 11]. This phenomenon was also observed in Korea, and the prevalence of myopia in a population of 19-year-old males was 96.5% and 93.3% in urban and rural areas of Korea, respectively [12, 13].

Research elucidating the pathogenesis of myopia and its risk factors would be necessary to prevent myopia and reduce the socioeconomic burden of the disease. Studies suggested that the risk of myopia was associated with parental myopia [14, 15], higher socioeconomic status [16–18], near work [11, 18], education [5, 12, 19], urban residence [1, 20], low serum vitamin D [4, 21], lesser outdoor activity [20, 22], and height [1, 19]. However, controversies exist among the studies, and the mechanism of myopia development remains unclear. Moreover, only few studies have been conducted regarding the risk factors of myopia in adult population.

In this study, we evaluated the prevalence and risk factors of myopia in a representative Korean adult population using the Korea National Health and Nutrition Examination Surveys (KNHANES), which is a series of nationwide cross-sectional health examination and survey conducted to monitor the general health and nutritional status of Koreans by the Division of Chronic Disease Surveillance, Korea Centers for Disease Control and Prevention (KCDC) [1].

## Patients and methods

### Study design and population

KNHANES are designed to produce a nationally representative data of the civilian, non-institutionalized Korean population [23]. KNHANES participants were sampled based on a stratified, multistage, cluster probability sampling, which uses the primary sampling units (PSU) as defined by the geographical areas in Korea [23]. In KNHANES VI that began in 2013, 192 enumeration districts were selected each year, and 20 households in each enumeration district were selected using a systematic sampling method [23].

KNHANES surveys consist of health interview survey, health examination survey, and nutrition survey. Information including age, sex, education level, education level of parents, annual income (quartile 1(lowest) to 4 (highest)), residential area (urban or rural), time spent on near work, occupational type, and parental myopia was obtained through health interviews. Anthropometric measurements were performed to yield variables, including height, weight, waist circumference and body mass index (BMI) calculated as weight (kg)/height^2^ (m^2^)[21]. Laboratory test results, including concentration of blood heavy metals, concentration of serum 25-hydroxyvitamin D (25(OH)D) concentration and complete blood count, were also collected.

The KNHANES was approved by the Institutional Review Board of the Korea Centers for Disease Control and Prevention (KCDC), and written informed consent was obtained from all participants in the survey. This study adhered to the tenets of the Declaration of Helsinki for biomedical research.

### Ophthalmologic examination and data collection

In KNHANES VI, participants aged 19 to 49 years were selected and received ophthalmological examinations, including visual acuity testing and autorefraction. Noncycloplegic autorefraction was performed using an autorefractor-keratometer (KR-8800, Topcon, Tokyo, Japan) in both eyes by trained ophthalmologists. Autorefraction measurements were converted into spherical equivalents (SE), as calculated by the spherical value + 1/2 of cylinder value. Refractive error was defined based on the left eye [1]. Myopia was defined as an SEQ ≤ -0.5 diopters (D), and non-myopia was defined as an SEQ >-0.5 D. Myopia was subdivided into low myopia (-6.0 D < SEQ ≤ -0.5 D) and high myopia (SEQ ≤ -6.0 D).

**Fig 1:**
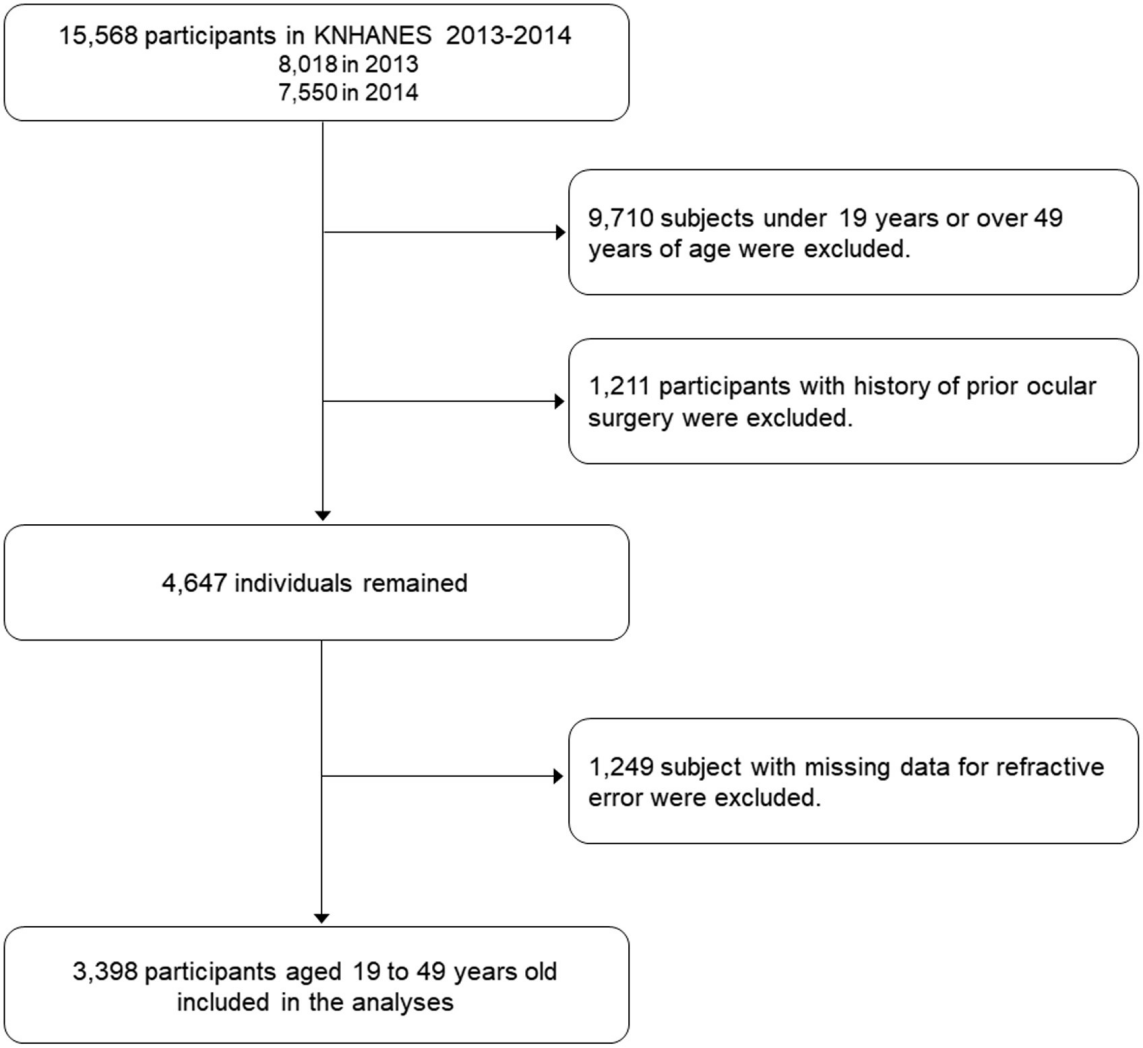
Flow diagram describing the selection of participants included in the present study.

Data from the KNHANES VI (VI -1, 2013 & VI—2, 2014) studies were used to investigate the prevalence and risk factors of myopia in adult Korean population. Among 15,568 (8,018 in 2013 and 7,550 in 2014) participants, 5,858 subjects who were aged between 19 and 49 years received an ophthalmic examination. Among them, 1,211 individuals with s history of prior ocular surgery, such as refractive surgery or cataract extraction, were excluded. Among the remaining 4,647 participants, 1,249 with missing data for refractive error were also excluded; 3,398 subjects were finally included in this study (Fig 1).

### Cut-points of serum 25(OH)D concentration

At first, we classified the serum 25(OH)D concentration to 10 percentiles. Then, we defined a group with the serum 25(OH)D concentration > 90 percentile as the reference group and calculated the odds ratios (ORs) for myopia by the serum 25(OH)D category. Among the nine groups, the 0–10 percentile group (serum 25(OH)D < 9 ng/mL) showed the strongest association with myopia prevalence. However, no significant increase in myopia prevalence by serum 25(OH)D of 11–20 percentile was observed. Thus, we determined the serum 25(OH)D concentration of 9 ng/mL as the cut-point.

### Categorization for time spent on near work

Responses to the question on time spent on near work were 1, 2, 3, and 4 hours/day in the KNHANES questionnaire. Because spending 3 or more hours/day on near work was significantly associated with increased myopia prevalence, we re-classified the four groups into two groups (≤2 hours/day and ≥3 hours/day) to ensure sufficient statistical power in stratified analysis by time spent on near work.

### Statistical analysis

KNHANES is derived from the multi-stage clustered probability sampling to produce a nationally representative survey data. To calculate the proper estimates and standard errors of the estimates, sampling components (strata and cluster), and appropriate weight should be considered in the analyses. Briefly, strata based on administrative district and housing type (apartment and conventional dwelling) and primary sampling unit (PSU), the cluster which consists of about 60 households, were used for sampling in KNHANES. Sample weight calculated by multiplying inverse of selection probability and inverse of response rate and adjusting them by reflecting age- and sex-specific population structure was also considered. PROC SURVEY procedures in the SAS program were developed for complex survey data analysis, thus we used these procedures in this work [24].

Continuous variables were presented using the weighted mean and standard deviation (SD) or median with interquartile range (IQR) in case of variables not distributed normally, and categorical variables were expressed as weighted frequencies with standard errors (SE) (%). The difference with respect to the means or the proportions of potential risk factors of myopia between non-myopia group and myopia group was assessed using t-test for normally distributed continuous variable, the Mann-Whitney U test for not normally distributed continuous variables, and Pearson’s Chi-square test for categorical variables.

The prevalence of myopia, low myopia, and high myopia in the total study population and in each age group was presented with weighted frequency (%) and SE. To calculate prevalence relative risks and their corresponding confidence intervals, we set the prevalence of myopia as dependent variable and age groups as independent variable in the logistic regression model.

Association between potential risk factors and prevalence of myopia (non-myopia (reference) vs. myopia) was evaluated by calculating the odds ratios (ORs) and 95% confidence intervals (CIs) using the binary logistic regression model. Independent variables included in the above binary logistic regression model were as follows: age, education level (< 12 vs. ≥ 12 years), parental myopia, serum 25(OH)D level (< 9 vs. ≥ 9 ng/mL), time spent on near work (≤2 vs. ≥3 hours/day), and white blood cell (WBC) counts (<5 x 10^3^, 5–8.9 x 10^3^, ≥9 x 10^3^). According to likelihood ratio test, logistic regression model including all independent variables above was the preferred model.

When it comes to analysis with consideration of myopia severity (low, and high myopia), binary logistic regression model was also used to assess the association between potential risk factors and low myopia (non-myopia (reference) vs. low myopia) or high myopia (non-myopia (reference) vs. high myopia).

Based on the results of multivariable analysis, the association between potential risk factors for the prevalence of total, high, and low myopia in a stratified group was also evaluated.

We set the type I error as 0.05 in this study. Statistical analyses were performed using SAS software (ver. 9.3; SAS Institute, Inc., Cary, NC, USA).

## Results

### General characteristics

The mean age of the study participants was 36.3 ± 8.6 (mean ± SD) years. The sex ratio was 54.5 ± 0.9% for male and 45.5 ± 0.9% for female, respectively. In univariable analysis, participants in the myopia group was significantly younger and on average 1 (95% CI, 0.3, 1.7) cm taller (*P* = 0.02), with lower serum 25(OH)D concentration than those in the non-myopia group. However, there was no significant difference in the height and serum 25(OH)D concentration between participants with low myopia and those with high myopia, although participants with high myopia were significantly younger than those with low myopia. The proportion of participants with parental myopia, high annual income, increased time of near work (≥3 hours/day), higher education level, and higher education levels of both parents were higher in the myopia group compared with the non-myopia group. Participants with high myopia were more likely to have parental myopia, increased time of near work, and higher education levels of both parents compared with those with low myopia (Table 1).

**Table 1:** General characteristics of study subjects aged 19–49 years old in the Korea National Health Examination and Nutritional Survey (KNHANES), Phase VI, 2013–2014.

Variables	Non-myopia (N = 1,025)	Myopia (N = 2,373)	*P*^1^	95% CI for difference^2^	Mild myopia (N = 2,112)	High myopia (N = 261)	*P*^1^	95% CI for difference^2^
	**Mean (SD)**	**Mean (SD)**			**Mean (SD)**	**Mean (SD)**		
Height (cm)	166.6 (8.8)	167.6 (8.7)	**0.02**	0.3–1.7	167.6 (8.7)	167.6 (8.8)	0.90	-1.3–1.3
Weight (Kg)	66.3 (12.7)	66.6 (13.6)	0.56	-0.7–1.3	66.5 (13.3)	67.4 (15.2)	0.46	-1.2–3.0
WC (cm)	80.0 (10.0)	79.7 (10.4)	0.52	-0.5–1.1	79.6 (10.3)	80.1 (11.3)	0.55	-1.1–2.1
BMI (Kg/m^2^)	23.8 (3.6)	23.6 (3.8)	0.29	-0.1–0.5	23.6 (3.7)	23.8 (4.3)	0.44	-0.4–0.8
	**Median (IQR)**	**Median (IQR)**			**Median (IQR)**	**Median (IQR)**		
Age (year)	39.5 (31.5–44.8)	33.8 (25.7–41.2)	**<0.01**	3.2–4.8	34.1 (26.2–41.5)	31.3 (22.8–39.5)	**<0.01**	0.7–3.9
Serum 25(OH) (ng/ml)	14.9 (11.6–19.0)	14.4 (11.3–18.1)	**0.02**	0.2–2.2	14.4 (11.2–18.1)	14.8 (11.3–18.1)	0.27	-1.0–1.4
Calcium intake (g/day)	449 (330–637)	445 (303–631)	0.23	-16–49	445.9 (303–634)	432.8 (303–600)	0.97	-58–59
Energy intake (Cal/day)	2005 (1521–2707)	2014 (1510–2688)	0.77	-96–99	2011 (1502–2689)	2069 (1605–2659)	0.20	-135–176
Hemoglobin (mg/dl)	14.4 (13.2–15.7)	14.6 (13.3–15.7)	0.19	-0.1–0.1	14.6 (13.3–15.7)	14.6 (13.3–15.8)	0.90	-0.2–0.2
WBC count (x10^3^)	6.1 (5.0–7.3)	6.1 (5.2–7.4)	0.01	-0.1–0.1	6.1 (5.2–7.3)	6.3 (5.5–7.7)	**<0.01**	0.0–0.6
	**N (%)**	**N (%)**			**N (%)**	**N (%)**		
Parental myopia	75 (8.3)	347 (16.1)	**<0.01**	5.6–10.0	281 (8.3)	66 (27.3)	**<0.01**	13.5–24.5
Female	599 (46.0)	1,293 (45.4)	0.76	-3.1–4.3	1,148 (45.3)	145 (45.6)	0.94	-6.1–6.7
Higher income ^3^	452 (43.1)	1,159 (47.9)	**0.03**	1.2–8.4	1,018 (47.5)	141 (51.2)	0.33	-2.7–10.1
Near work ≥ 3 hour/day	449 (45.2)	1,438 (63.5)	**<0.01**	14.7–21.9	967 (48.3)	147 (57.4)	**<0.01**	5.5–16.9
Education ≥ 12 years	372 (39.4)	1,143 (49.3)	**<0.01**	6.3–13.5	1,000 (48.7)	143 (54.1)	0.11	-1.0–11.8
Father’s education ≥12 years	746 (88.5)	1,666 (78.7)	**<0.01**	7.2–12.4	1,502 (80.3)	164 (66.6)	**<0.01**	7.7–19.7
Mother’s education ≥12 years	826 (95.8)	1,894 (89.7)	**<0.01**	4.4–7.8	1,699 (90.7)	195 (81.5)	**<0.01**	4.3–14.1

WC, Waist Circumference; BMI, Body mass index; WBC, White blood cell.

All data are expressed as mean (SD), median (interquartile range (IQR)) or weighted frequency

^1^. P value using independent t-test or Mann-Whitney test for continuous variable, and Pearson’s chi-square test for categorical variable

^2^. 95% confidence intervals for difference in mean or proportion of each variables

^3^. Above median annual household income in Korean population

### Prevalence of myopia

The prevalence of myopia and high myopia were 70.6 ± 1.1% and 8.0 ± 0.6%, respectively, which decreased significantly with age, from 81.3 ± 0.8% and 13.3 ± 0.4% in the population aged 19 to 24 years to 55.2 ± 0.7% and 4.0 ± 0.2% in the population aged 45 to 49 years, respectively (Table 2).

**Table 2:** Prevalence of myopia in various age groups.

Age (year)	Total	Myopia*	Low myopia	High myopia	Myopia*	Low myopia	High myopia
	N	N, Prevalence (%±SE)	N, Prevalence (%±SE)	N, Prevalence (%±SE)	PRR (95% CI) ^†^	PRR (95% CI) ^†^	PRR (95% CI) ^†^
Total	3,398	2,373 (70.6±1.1)	2,112 (62.6±1.0)	261 (8.0±0.6)			
19–24	463	383 (81.3±0.8)	325 (68.0±0.7)	58 (13.3±0.4)	**3.53 (2.54, 4.92)**	**3.19 (2.30, 4.42)**	**7.96 (4.24, 14.95)**
25–29	339	269 (79.6±0.7)	238 (70.5±0.7)	31 (9.1±0.2)	**3.17 (2.22, 4.53)**	**3.02 (2.10, 4.35)**	**5.00 (2.67, 9.35)**
30–34	508	390 (76.9±0.7)	346 (68.9±0.7)	44 (8.0±0.2)	**2.70 (2.01, 3.65)**	**2.61 (1.93, 3.54)**	**3.86 (2.19, 6.80)**
35–39	664	472 (71.0±0.7)	425 (64.3±0.6)	47 (6.7±0.2)	**1.99 (1.57, 2.52)**	**1.94 (1.54, 2.46)**	**2.58 (1.47, 4.53)**
40–44	737	478 (65.3±0.7)	423 (57.7±0.6)	55 (7.6±0.2)	**1.53 (1.21, 1.94)**	**1.46 (1.15, 1.85)**	**2.45 (1.43, 4.20)**
45–49	687	381 (55.2±0.7)	355 (51.2±0.6)	26 (4.0±0.2)	1.00	1.00	1.00

SE, standard error; PRR, prevalence relative risk

*Myopia was divided into low myopia and high myopia.

^†^Calculated based on the comparison with prevalence of non-myopia, with age group of 45–49 year as the reference level

**Fig 2:**
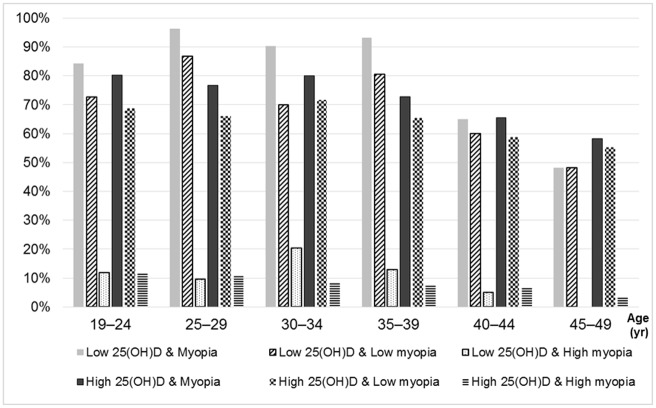
Prevalence of myopia, low myopia and high myopia by various age groups in two stratified groups according to serum 25(OH)D concentration (low 25 (OH)D = serum 25(OH)D concentration < 9 ng/mL; high low 25 (OH)D = serum (OH)D concentration ≥ 9 ng/mL).

Myopia prevalence according to age in the stratified group by serum 25(OH)D level (< 9 *vs*. ≥ 9 ng/mL) is shown in Table 3 and Fig 2. The prevalence of myopia, low myopia, and high myopia in the low serum 25(OH)D group (82.0%, 71.7%, and 10.3%, respectively) were higher than the high serum 25(OH)D group (72.5%, 64.3%, and 8.2%, respectively). The prevalence of myopia, low myopia, and high myopia tended to be higher in the low serum 25(OH)D group compared with the high serum 25(OH)D group in each age group, particularly in younger age groups. Regardless of the serum 25(OH)D level, the group aged from 45 to 49 years was associated with the lowest prevalence of myopia compared with other age groups.

**Table 3:** Prevalence of myopia by various age groups in two stratified groups according to serum 25(OH)D concentration.

Age (year)	Serum 25(OH)D concentration < 9 ng/mL				Serum 25(OH)D concentration ≥ 9 ng/mL			
	Total	Myopia*	Low myopia	High myopia	Total	Myopia*	Low myopia	High myopia
	N	N, Prevalence (%±SE)	N, Prevalence (%±SE)	N, Prevalence (%±SE)	N	N, Prevalence (%±SE)	N, Prevalence (%±SE)	N, Prevalence (%±SE)
Total	141	114 (82.0±0.4)	100 (71.7±0.4)	14 (10.3±0.1)	1,373	991 (72.5±0.9)	881 (64.3±0.9)	110 (8.2±0.4)
19–24	38	32 (84.4±4.0)	27 (72.7±3.8)	5 (11.7±1.6)	234	193 (80.2±1.3)	167 (68.6±1.2)	26 (11.5±0.6)
25–29	27	26 (96.4±4.2)	23 (86.8±4.2)	3 (9.6±1.3)	178	138 (76.7±1.2)	120 (66.0±1.1)	18 (10.6±0.4)
30–34	21	18 (90.3±3.1)	14 (69.9±2.8)	4 (20.4±1.6)	199	155 (80.0±0.9)	137 (71.6±0.9)	18 (8.4±0.3)
35–39	15	14 (93.2±2.1)	13 (80.4±1.8)	1 (12.8±1.0)	262	192 (72.8±0.8)	171 (65.4±0.8)	21 (7.5±0.3)
40–44	21	14 (65.0±2.0)	13 (60.0±1.9)	1 (5.0±0.6)	270	177 (65.6±0.8)	157 (58.7±0.8)	20 (6.9±0.3)
45–49	19	10 (48.2±2.1)	10 (48.2±2.1)	0 (0.0±0.0)	230	136 (58.2±0.8)	129 (55.2±0.8)	7 (3.0±0.2)

SE, standard error

*Myopia was divided into low myopia and high myopia.

### Factors associated with myopia

The results of multivariable logistic regression to elucidate potential risk factors of myopia are summarized in Table 4. Younger age, education level ≥12 years, parental myopia, lower serum 25(OH)D concentration (<9 ng/mL), greater near work of ≥ 3 hours/day and WBC counts of 5,000 to 8,999 were significantly associated with increased myopia prevalence. Subjects aged 19 to 29 years and those aged 30 to 39 years showed 2.11-fold (95% CI, 1.64, 2.73) and 1.59-fold (95% CI, 1.31, 1.94) increased prevalence of myopia compared with those aged 40 to 49 years, respectively (Table 4).

**Table 4:** Association with potential risk factors for the likelihood of myopia among adults aged between 19 and 49 years in the Korea National Health Examination and Nutritional Survey (KNHANES) Phase VI, 2013–2014.

Variables	Non-myopia (N = 1,025) N	Myopia (N = 2,373) N	OR (95% CI)^1^
Age (years)			
19–29	150	652	**2.11 (1.64, 2.73)**
30–39	310	862	**1.59 (1.31, 1.94)**
40–49	565	859	1.00
Education level (years)			
<12	555	1,047	1.00
12+	372	1,143	**1.49 (1.23, 1.80)**
Parental myopia			
No	885	1,888	1.00
Yes	75	347	**1.55 (1.12, 2.13)**
Serum 25(OH)D (ng/ml)			
<9	27	114	1.00
9+	382	991	0.62 (0.38, 1.01)
Near work (hours/day)			
≤2	576	935	1.00
3+	449	1,438	**1.52 (1.28, 1.81)**
WBC counts x10^3^			
<5	251	453	1.00
5–8.9	645	1,622	**1.26 (1.01, 1.56)**
9+	64	151	1.11 (0.75, 1.65)

^1^. Adjusted for age, educational levels, parental myopia, serum 25(OH)D levels, time spent on near work, and WBC count

In a subgroup analysis by severity of myopia (low myopia and high myopia), younger age, higher education level (≥ 12 years), parental myopia, and longer time spent on near work were significantly associated with elevated prevalence of low myopia (Table 5). Participants aged 19 to 29 years and 30 to 39 years showed 2.04-fold (95% CI, 1.58, 2.63) and 1.62-fold (95% CI, 1.32, 1.98) increased low myopia prevalence compared with those aged 40 to 49 years, respectively. Near work of ≥ 3 hours/day was associated with increased prevalence of low myopia compared with near work of ≤2 hours/day (OR, 1.48; 95% CI, 1.24, 1.77).

**Table 5:** Association with potential risk factors for the likelihood of high and low myopia relative to non-myopia among adults aged between 19 and 49 years in the Korea National Health Examination and Nutritional Survey (KNHANES) Phase VI, 2013–2014.

Variables	Non-myopia N	Low myopia N	OR (95% CI)^1^	Non-myopia N	High myopia N	OR (95% CI)^1^
Age (years)						
19–29	150	563	**2.04 (1.58, 2.63)**	150	89	**2.55 (1.62, 4.02)**
30–39	310	771	**1.62 (1.32, 1.98)**	310	81	1.37 (0.92, 2.02)
40–49	565	778	1.00	565	81	1.00
Education level (years)						
<12	555	943	1.00	555	104	1.00
12+	372	1,000	**1.44 (1.19, 1.74)**	372	143	**1.80 (1.30, 2.49)**
Parental myopia						
No	885	1,706	1.00	885	182	1.00
Yes	75	281	**1.41 (1.01, 1.95)**	75	66	**3.11 (2.02, 4.77)**
Serum 25(OH)D (ng/ml)						
<9	27	100	1.00	27	14	1.00
9+	382	881	0.63 (0.38, 1.03)	382	110	**0.49 (0.25, 0.97)**
Near work (hours/day),						
≤2	576	856	1.00	576	79	1.00
3+	449	1,256	**1.48 (1.24, 1.77)**	449	182	**2.17 (1.55, 3.04)**
WBC counts x10^3^						
<5	251	418	1.00	251	35	1.00
5–8.9	645	1,437	1.23 (0.98, 1.53)	645	185	**1.75 (1.04, 2.94)**
9+	64	125	1.00 (0.67, 1.49)	64	26	**2.69 (1.28, 5.66)**

^1^. Adjusted for age, educational levels, parental myopia, serum 25(OH)D levels, time spent on near work, and WBC count

Increased prevalence of high myopia showed a significant association with younger age, higher education level (≥ 12 years), parental myopia, lower serum 25(OH)D concentration (<9 ng/ml), longer time of near work, and greater WBC count (≥ 5,000). The young age group (age from 19 to 29 years) showed elevated prevalence of high myopia (OR, 2.55; 95% CI, 1.62, 4.02) compared with the group aged 40 to 49 years. Near work of ≥ 3 hours daily had a 2.17-fold (95% CI, 1.55, 3.04) increased prevalence of high myopia than near work of ≤2 hours daily (Table 5).

The association of the serum 25(OH)D concentration with total, low, and high myopia in a stratified group for time spent on near work (≤2 hours/day *vs*. ≥3 hours/day) are shown in Table 6. In subjects with near work of ≤2 hours, no significant influences of serum 25(OH)D on total, low, and high myopia were observed. In participants with near work of ≥3 hours/day, serum 25(OH)D concentration of ≥9 ng/ml was significantly related to decreased prevalence of high myopia, although the effect was not significant in myopia and low myopia.

**Table 6:** 2014 The association of the serum 25(OH)D concentration with total, low, and high myopia in two strata for time spent on near time work ≤2 hours/day vs. ≥3 hours/day) among adults at age 19–49 years old in the Korea National Health Examination and Nutritional Survey (KNHANES) Phase VI, 2013–2014.

Variables	Non-myopia N	Myopia N	OR (95% CI)^1^	Non-myopia N	Low myopia N	OR (95% CI)^1^	Non-myopia N	High myopia N	OR (95% CI)^1^
Near work ≤ 2 (hour/day)									
Serum 25(OH)D (ng/ml)									
<9	11	33	1.00	11	31	1.00	11	2	1.00
9+	211	373	0.90 (0.36, 2.20)	211	345	0.87 (0.35, 2.15)	211	28	1.45 (0.21, 9.98)
Near work ≥ 3 (hour/day)									
Serum 25(OH)D (ng/ml)									
<9	16	81	1.00	16	69	1.00	16	12	1.00
9+	171	618	0.44 (0.19, 1.05)	171	536	0.47 (0.19, 1.13)	171	82	**0.28 (0.10, 0.80)**

^1^. Adjusted for age, educational levels, parental myopia, and WBC counts

## Discussion

The results of this study revealed a high prevalence of myopia and high myopia, 70.6± 1.1% and 8.0 ± 0.6%, respectively, in Korean adults aged between 19 and 49 years. This prevalence was shown to decrease with age. A study using the KNHANES data of the year 2008–2011 showed a sharp decline in the prevalence of myopia with increasing age; from 78.9% in participants aged 20–29 years to 16.1% in those aged 60–69 years [25]. These findings suggest that the prevalence of myopia might be higher in younger age groups, which can possibly contribute to the overall increase in the prevalence of myopia.

In Caucasians, the prevalence of myopia is substantially lower compared with the results of studies using KNHANES data including ours [25, 26]. Using the US NHANES data of 1999–2004, Vitale et al [2]. reported that the prevalence of myopia of ≤ -1.0 D in a population of ≥ 20 year was 33.1%. In the Los Angeles Latino Eye Study, myopia prevalence was 16.8% in adults of ≥ 40 years old [27]. A British study showed that the prevalence of myopia of ≤ -0.75 D was 49% in the 44 year old birth cohort [28]. In Norway, myopia prevalence was 35.0% in the group of 20–25 years old [29].

Higher prevalence of myopia in East Asians, especially those in urban areas, compared with Caucasians has been reported [30]. In Singapore, the prevalence of myopia of ≤ -0.5 D in Malay, Indian and Chinese adults > 40 years was 26.2% [8], 28.0% [9], and 38.7% [7], respectively [7–9], suggesting that myopia might be more prevalent in Chinese than in other ethnic groups. The prevalence of myopia in Japanese adults between the ages of 40 and 79 years was 45.7% in men and 38.3% in women [16]. Another study showed that the prevalence of myopia of ≤ -0.5 D was 41.8% in Japanese adults ≥ 40 years [10]. The results of this study, as well as those of previous studies using KNHANES data, suggest that the prevalence of myopia in Korean adults might increase more sharply compared with those in other countries [25, 26, 31].

Multivariable logistic regression analysis revealed that both myopia and high myopia had significant association with younger age, higher education level, parental myopia, lower serum vitamin D concentration, increased time of near work, and higher WBC counts, though the association between low serum 25(OH)D and myopia prevalence was marginally significant. Previous studies using KNHANES data revealed that younger age, higher education level, lower serum vitamin D levels, and shorter daily sun exposure may be risk factors for myopia [25, 26, 31]. The results of the present study are consistent with those of prior studies, and also suggest the association between WBC counts and both myopia and high myopia.

Several studies demonstrated the association between parental myopia and the risk of myopia [11, 14, 15, 18, 32–36]. Low et al. [14] indicated that a family history of myopia might be the most important risk factor for early myopia. Parental myopia was suggested to influence the growth rate of children’s eye [15, 37]. Saw et al. [35] revealed that children with myopic parents tended to have increased axial length.

In this study, near work and higher education level were associated with both myopia and high myopia. A British study also revealed this association of myopia with near work and educational performance [28]. Several studies also indicated that near work including reading and computer use, may increase the risk of myopia [9, 11, 32, 38–40]. Saw et al [35]. reported that children who read > 2 books a week had longer axial lengths by 0.17 mm compared with those who read ≤2 books a week. Previous studies also suggested that myopia may be associated with higher educational level [7, 8, 16, 18, 19, 25, 27, 30, 41] or higher academic achievements [12, 13, 32]. Education level can be considered a surrogate for near work, as both the achievement and duration of education are conceivably closely correlated with time spent studying and reading [42]. A meta-analysis suggested that lower education level could attenuate the influence of risk alleles on myopia, which underscores the role of gene-environment interactions in the development of myopia [43]. High hereditability of myopia does not preclude strong environmental influence, and the environmental impact can increase the risk of myopia across the population to a similar extent, which results in rapid change in the prevalence of myopia [44]. For instance, due to environmental pressures, including education and near work in Singapore, a large number of children with non-myopic parents have high myopia.[37]. A sharp increase in the prevalence of myopia in younger age groups in Korea may also reflect this phenomenon.

In the present study, the low serum 25(OH)D concentration was also shown to be related to increased prevalence for both myopia and high myopia, which is consistent with previous studies using the KNHANES data [21, 31]. Other researchers also revealed that myopes had a significantly lower blood vitamin D concentration compared with non-myopes [4, 45], A large population-based study revealed that polymorphisms within vitamin D receptor (VDR) are related to a low-to-moderate degree of myopia [46]. As endogenous synthesis of vitamin D is induced by sunlight, it is often considered a biomarker of outdoor activity [46]. The prevalence of vitamin D insufficiency has been increasing worldwide [47, 48]. Data from KNHANES also showed that vitamin D deficiency was highly prevalent in Korean adolescents [49], which can be explained by reduced sunlight exposure caused by high educational pressure and decreased intake of vitamin D-rich foods [49, 50]. Given that vitamin D plays an important role in the intestinal absorption of calcium, mineral homeostasis, and bone growth in adolescence [51], it can be hypothesized that vitamin D deficiency may be associated with the development of myopia in adolescence [51]. Moreover, vitamin D deficiency can also result in the impairment of relaxation and contraction of the ciliary muscles caused by an alteration of intracellular calcium concentration, which may lead to myopia genesis [52]. Moreover, vitamin D initiates the formation of the VDR/retinoic acid heterodimer [53], which participates in the retinoscleral signal pathway that may influence myopic ocular growth [21]. The major product of novel cytochrome P450scc (CYP11A1)-initiated pathway of vitamin D_3_ metabolism, 20-hydroxyvitamin D_3_ (20(OH)D_3_), and its metabolites may have anti-proliferative, pro-differentiation, and anti-inflammatory effects, which could prevent myopia [54–56].

Several studies demonstrated the protective effect of outdoor activity in myopia development [20, 22, 57–62]. Although the mechanisms underlying the possible protective effect of outdoors activity is unclear, there have been two hypotheses to date: 1) Higher light intensity of the outdoors may stimulate retinal dopamine release [22], which was proven to suppress axial elongation in animal model [63]. 2) Brighter outdoor light intensity may increase the depth of focus through pupil constriction, leading to reduced accommodative demand [22, 57]. These may not directly be applicable to adults with full eyeball maturity [22]. The most plausible explanation would be that the results reflect a continuation in the occupation or behavior. Individuals who spend more time reading books during childhood are more likely to become office workers and be involved in work related to reading and using the computer [22].

Regarding the relationship between vitamin D and outdoor activity, Kwon et al [31]. showed that both factors may be independently associated with myopia in Korean adults. However, a cohort study demonstrated that there was no association between vitamin D and myopia, while outdoor activity had protective effect on myopia [64]. By contrast, another study revealed that the association between vitamin D and myopia retained after adjustment for conjunctival UV autofluorescence, an ocular sun-exposure biomarker [4]. We believe that further studies are needed to investigate the pathophysiology underlying the possible influence of vitamin D and outdoor activity on myopia [4].

Among the risk factors for myopia found in the present study, only serum 25(OH)D concentration and time of near work are controllable. Considering the high pressure for education and increased computer use, reducing the time of near work would be difficult. Given the possible association between higher serum 25(OH)D concentration and decreased risk of high myopia in adults with increased time of near work, it could be possible to prevent high myopia by increasing serum prevented concentration, possibly by vitamin D supplementation or vigorous outdoor activities.

Increased WBC counts were also associated with both myopia and high myopia, indicating the relationship between inflammatory response and myopia progression. Although only few studies have studied the association between myopia and inflammation, Lin et al.[65] showed that the inhibition of inflammatory response delayed myopia development and aggravation of inflammation accelerated myopia progression. We believe further studies are necessary to elucidate the influence of inflammation on myopia.

The present study has some limitations. First, refractive error was measured without cycloplegia, which could lead to the overestimation of the prevalence of myopia. However, this is a limitation shared by most of the studies on refractive errors in adults, as performing a cycloplegic refraction in a large population is almost impossible. Second, due to the cross-sectional design, a causal relationship could not be evaluated. We believe further prospective studies are needed for the evaluation of the effect of proposed risk factors of myopia. Third, this study did not evaluate the time of outdoor activity, serum calcium level, and bone parameters. Therefore, the potential protective effect of vitamin D supplementation or outdoor activity was never evaluated. Further studies including these variables are therefore warranted. Fourth, participants with a history of ocular surgery were excluded. However, considering that this study included patients aged 19 to 49 years, a substantial number of excluded subjects possibly have history of vision correction surgery for myopia, which could lead to underestimation of myopia prevalence.

In conclusion, this study showed a high prevalence of myopia and high myopia in Korean adults, which increased in younger age groups. In addition to parental myopia, low serum 25(OH)D concentration and near work may be associated with myopia. Inflammation reflected by WBC counts might also be related to myopia.
